# B Lymphocyte—A Prognostic Indicator in Post‐Acute Pancreatitis Diabetes Mellitus

**DOI:** 10.1111/1753-0407.70047

**Published:** 2025-01-12

**Authors:** Xiaoyan Lin, Xiaoling Li, Junsheng Wang, Huiheng Liu

**Affiliations:** ^1^ Clinical Laboratory Center of Zhongshan Hospital Xiamen University Xiamen China; ^2^ School of Medicine, Xiamen University Xiamen China; ^3^ Emergency Department of Zhongshan Hospital Xiamen University Xiamen China

**Keywords:** B%, CD10, HLA‐DR, lymphocytes, MFI, PPDM‐A, PPNG‐A, risk factors

## Abstract

**Objective:**

To determine the value of lymphocyte subsets and granulocyte/monocyte surface markers in predicting the risk of post‐acute pancreatitis diabetes (PPDM‐A).

**Methods:**

This study included 308 in patients with acute pancreatitis (AP). The markers of granulocytes and monocytes and lymphocyte subsets were detected by flow cytometry, and the fluorescence intensity, absolute count and percentage were obtained. Based on the occurrence of diabetes after AP, patients were divided into two groups: PPDM‐A and PPNG‐A (post‐acute pancreatitis with normal glucose). Correlations between granulocyte and monocyte surface markers and lymphocyte subsets were analyzed. Binary logistic regression was used to analyze the potential influencing factors of PPDM‐A.

**Results:**

Compared with patients with PPNG‐A, patients with PPDM‐A tend to be younger (*p* < 0.001) and have a higher proportion of fatty liver, recurrent pancreatitis, and hyperlipidemic pancreatitis. The results of linear regression showed that B% was negatively correlated with MFI of HLA‐DR on monocytes (*R* ^2^ = 0.145, *p* < 0.001), B% was positively correlated with CD10^−^NEUT% (*R* ^2^ = 0.291, *p* < 0.001), and MFI of HLA‐DR on monocytes was negatively correlated with CD10^−^NEUT% (*R* ^2^ = 0.457, *p* < 0.001). Multivariate logistic regression analysis revealed that age, serous effusion, fatty liver, recurrent pancreatitis, and B% were independent risk factors for the occurrence of PPDM‐A.

**Conclusion:**

Our study has first confirmed the correlation between PPDM‐A and lymphocyte subsets and CD10^−^NEUT%. Furthermore we indicated that age, fatty liver, serous effusion, recurrent AP, and B% were independent risk factors for PPDM‐A. The mechanism of granulocyte and monocyte surface markers and B lymphocytes on PPDM‐A is worthy of study. This would help clarify the pathogenesis of PPDM‐A at the cellular level and potentially provide new strategies for immunotherapy and even disease prevention. [Correction added on 24 January 2025, after first online publication: the third subtitle in Abstract section has been changed to ‘Results’.]

## Graphical abstract

In this study, we aimed to determine the value of lymphocyte subsets and granulocyte/monocyte markers in predicting the risk of post‐acute pancreatitis diabetes mellitus (PPDM‐A). Our study is the first to confirm the correlation between PPDM‐A and lymphocyte subsets and indicated that age, fatty liver, serous effusion, recurrent AP, and B% were independent risk factors for PPDM‐A. The interaction mechanism of granulocyte and monocyte surface markers and B lymphocytes on glucose metabolism disorder after AP is worthy of further study.

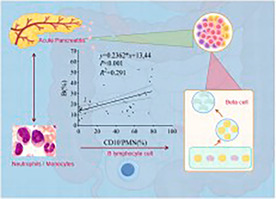

**Abbreviations**
AP: acute pancreatitis
DEP: pancreatic exocrine diabetes mellitus
DNT: double negetive T lymphocyte
DPT: double positive T lymphocyte
LYM: lymphocyte
MAP: mild acute pancreatitis
MDSC: myeloid‐derived suppressor cell
MFI: mean fluorescence intensity
MONO: monocyte
MSAP: moderately severe acute pancreatitis
NEUT: neutrophils
Non‐MAP: non‐mild acute pancreatitis
PPDM‐A: post‐acute pancreatitis diabetes
PPNG‐A: post‐acute pancreatitis with normal glucose
SAP: severe pancreatitis

**Summary**

- Our study has first confirmed the correlation between PPDM‐A and lymphocyte subsets and CD10^−^NEUT%.
- Furthermore we indicated that age, fatty liver, serous effusion, recurrent AP, and B% were independent risk factors for PPDM‐A.
- The mechanism of granulocyte and monocyte surface markers and B lymphocytes on PPDM‐A is worthy of study.
- This would help clarify the pathogenesis of PPDM‐A at the cellular level and potentially provide new strategies for immunotherapy and even disease prevention.

## 1 Introduction

Acute pancreatitis (AP) is a common cause of acute abdominal pain in the gastrointestinal tract, characterized by acute aseptic inflammation of the pancreas and destruction of acinar cells. Diabetes is a heterogeneous disease with various clinical features [1]. Pancreatic exocrine diabetes mellitus (DEP) is a type of secondary diabetes caused by pancreatic exocrine dysfunction. Since AP is currently the most common disease affecting the exocrine part of the pancreas, it may also be the most common cause of DEP [2]. Survivors of severe acute pancreatitis (SAP) may experience permanent end‐organ dysfunction, including post‐pancreatitis diabetes mellitus (PPDM) [3]. It had been reported that 23% of patients with AP develop new‐onset diabetes. However, few people pay attention to the history of pancreatitis, and only half of DEP patients are correctly diagnosed, with the remainder often misclassified as either type 2 diabetes (T2DM) or type 1 diabetes (T1DM) [4]. The original severity score for AP included hyperglycemia as a risk factor for serious illness, but the most recent APACHE II score did not [3]. The relationship between diabetes and AP is complex, and the influencing factors are not yet fully understood.

AP is often accompanied by elevated blood glucose levels, which occurs with the involvement of the nervous system and is also regulated by hormones other than insulin. Insulin secretion is also abnormal due to changes in the islet microenvironment [5]. During AP, oxidative stress in the mononuclear macrophage system is enhanced, which not only reduces insulin secretion but also promotes insulin resistance and gluconeogenesis. The abnormal increase in blood glucose during SAP significantly disrupts the body's internal environment, weakens its ability to resist pancreatic and other infections, and creates a vicious cycle with islet antagonism caused by infection. Clinical studies have indicated that one way to reduce complications and mortality in SAP is to use intensive insulin therapy to effectively control glucose metabolism disorders [6]. Therefore, the presence of hyperglycemia after AP reflects the severity of the condition to some extent; in SAP, the longer the duration, the greater the risk of glucose metabolism disorders in the later stages [6].

Over the years, the medical community has increasingly recognized that immune events play a critical role in determining the course of diseases [7]. Recently, the activation of the immune system has been identified as a key trigger and regulator of AP injury, affecting the extent of pancreatic necrosis, organ failure, and disease progression [8]. Lymphocytes are a type of white blood cell with specialized immune functions, including T lymphocytes involved in cellular immunity, B lymphocytes involved in humoral immunity, and NK cells that can directly kill target cells [9]. The maintenance of normal immune function in the human body depends on the proper proportion of TBNK cell subsets. Lymphocyte subsets play an important role in mediating and regulating the immune pathogenesis of T1DM [10]. The role of B cells in diabetes is particularly complex. B cells serve as key antigen‐presenting cells (APCs) in the process of islet cell injury mediated by T lymphocytes [11], and they also secrete autoantibodies. When β‐cell autoantigens are exposed, B lymphocytes become activated and secrete insulin autoantibodies (IAA) and other autoantibodies [12]. Previous studies have also suggested that the number of Breg cells producing IL‐10 in the peripheral blood of diabetic mice decreases, and that the frequency of B cells in the marginal zone of the spleen changes. The imbalance in the frequencies of these lymphocyte subsets may be associated with the development of diabetes [13]. However, data on whether the characteristics of insulin resistance in post‐acute pancreatitis diabetes (PPDM‐A) are related to B cells remain scarce. Inspired by the aforementioned evidence, our study aims to describe the differences in granulocyte and monocyte surface markers, as well as lymphocyte subsets, between the PPDM‐A and post‐acute pancreatitis with normal glucose (PPNG‐A) groups, and to determine whether there is a correlation between lymphocyte subsets and granulocyte or monocyte surface markers. Our research could offer new insights into the immune characteristic differences between PPDM‐A and PPNG‐A patients.

The diagnosis of AP requires the presence of two out of the following three criteria [14]: (i) abdominal pain consistent with AP (persistent and severe epigastric pain, often radiating to the back); (ii) serum lipase activity (or amylase activity) at least three times greater than the upper normal limit; and (iii) typical findings of AP on contrast‐enhanced computed tomography (CECT), magnetic resonance imaging (MRI), or transabdominal ultrasound.

PPDM‐A: It should be diagnosed after excluding pre‐existing diabetes and using the American Diabetes Association (ADA) criteria 90 days after the onset of AP. The criteria include glycosylated hemoglobin (HbA1c) ≥ 48 mmol/mol or 6.5% and/or fasting blood glucose > 7 mmol/L or 126 mg/dL.

PPNG‐A: Post‐acute pancreatitis with normal glucose. That is, non‐PPDM‐A.

## 2 Methods

### 2.1 Study Population

**FIGURE 1:**
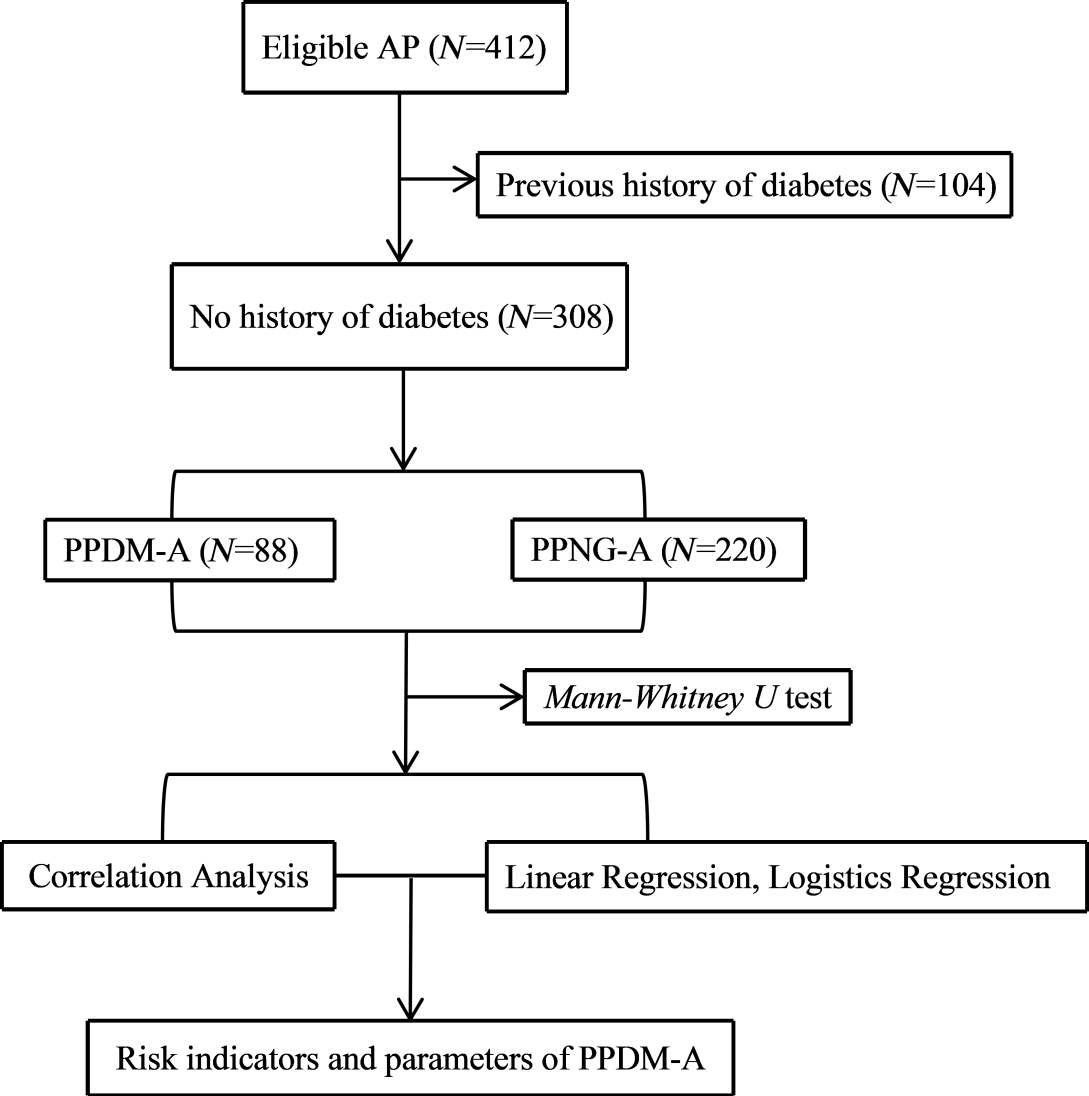
The study flow chart.

This was a prospective study. The selected cases were patients with AP who were admitted to Zhongshan Hospital Xiamen University, from April 2023 to April 2024. The inclusion criteria were patients who met the definition of AP. The exclusion criteria were as follows: [1] age less than 18 years or greater than 80 years; [2] cases of chronic pancreatitis or acute exacerbation of chronic pancreatitis; [3] discharge within 4 days; [4] patients with hematologic diseases, solid tumors, chronic respiratory failure, chronic renal failure, autoimmune diseases, or other infectious diseases; and [5] incomplete clinical data. A total of 412 patients were included, of which 104 had diabetes prior to the onset of AP, and 308 had no previous history of diabetes (220 males and 88 females). Based on the occurrence of PPDM‐A, the patients were divided into two groups: PPDM‐A and PPNG‐A. The study flow chart is shown in Figure 1.

### 2.2 Data Sources and Detection Methods

Demographic parameters (sex, age), length of hospital stay, medical history, severity of AP, etiological factors, complications, infection status, and treatment plan were obtained through the hospital information system (HIS). Surface markers and lymphocyte subsets of granulocytes and monocytes were detected using the Mindray BriCyte E6 flow cytometer, with fluorescence intensity, absolute count, and percentage recorded. Serum amylase (AMY), lipase (LPS), and blood glucose (GLU) levels were measured using the Beckman AU5800 automatic biochemical analyzer, while glycosylated hemoglobin (HbA1c) was assessed using the ARKRAY HA 8180 glycosylated hemoglobin analyzer from Japan.

### 2.3 Statistical Methods

Statistical comparisons were performed using the IBM SPSS 27.0 Statistics package (IBM, USA). Continuous variables were expressed as unit number (N), percentage (%), and median/range (*M, IQR*). The Mann–Whitney *U* test and *χ* ^2^ *test* were used to compare differences in parameters between the PPDM‐A and PPNG‐A groups, analyze the results of granulocyte and monocyte surface markers and lymphocyte subsets in patients with PPDM‐A and PPNG‐A, and identify the parameters with the greatest differences between groups. The *Spearman test* was used to detect correlations between variables. Linear regression was applied to analyze the linear relationship of continuous variables, while multivariate logistic regression was used to analyze potential risk factors for PPDM‐A. All tests were two‐sided, and *p* < 0.05 was considered statistically significant.

## 3 Results

### 3.1 Characteristics of the Study Population

The demographic and clinical characteristics of the patients are shown in Table 1. According to the revised Atlanta criteria, 147 patients (47.7%) were diagnosed with mild acute pancreatitis (MAP), while 161 patients (52.3%) were diagnosed with non‐MAP. The patients were divided into two groups based on the occurrence of PPDM‐A: patients with PPDM‐A (*n* = 88) and patients without PPDM‐A (PPNG‐A, *n* = 220). There were 155 males and 65 females in the PPNG‐A group, and 65 males and 23 females in the PPDM‐A group. The median age of the PPDM‐A group was 42 years, while that of the PPNG‐A group was 52 years. In the PPNG‐A group, the proportion of biliary pancreatitis was similar to that of hyperlipidemic pancreatitis, whereas in the PPDM‐A group, the proportion of MAP and non‐MAP was 38.6% and 61.4%, respectively, with a significantly higher proportion of non‐MAP in PPDM‐A cases. Compared with the PPNG‐A group, the PPDM‐A group had a higher proportion of fatty liver and a higher incidence of recurrent AP.

**TABLE 1:** Demographic data, etiology, and comorbidity records of cases in AP study groups.

		PPNG‐A	PPDM‐A
Number of patients (*N*)		220	88
Gender			
	Male	155 (70.5%)	65 (73.9%)
	Female	65 (29.5%)	23 (26.1%)
Median age (M, IQR)		52, 27	42, 14
Etiology			
	Biliary	57 (25.9%)	5 (5.7%)
	Hyperlipidemic	70 (31.8%)	64 (72.7%)
	Alcoholic	38 (17.3%)	0 (0.0%)
	Mixed type	39 (17.7%)	19 (21.6%)
	Idiopathic	16 (7.3%)	0 (0.0%)
Severity			
	MAP	113 (51.4%)	34 (38.6%)
	Non‐MAP	107 (48.6%)	54 (61.4%)
Complication			
	Effusion in the serous cavity	88 (40.0%)	58 (65.9%)
	Hypertension	82 (37.3%)	22 (25.0%)
	Fatty liver	89 (40.5%)	65 (73.9%)
	Infection	48 (21.8%)	6 (6.8%)
Prognostic indicator			
	Recurrent attack AP	57 (25.9%)	70 (79.5%)
	Intensive care unit stay	50 (22.7%)	20 (22.7%)
	Deterioration of condition/dead	20 (9.1%)	6 (6.8%)
	Mechanical ventilation	23 (10.5%)	0 (0.0%)
	Continuous renal replacement therapy	38 (17.3%)	20 (22.7%)

### 3.2 The Comparisons of Surface Expression of Neutrophils/Monocytex and Lymphocyte Subsets Between PPNG‐A and PPDM‐A Groups

By comparing the ages between the two groups, we can conclude that the PPDM‐A group is younger than the PPNG‐A group, with the difference being statistically significant (*p* < 0.05). Additionally, there were significant differences in the CD64 index, CD10^−^NEUT (%), CD10^+^NEUT (%), total lymphocyte count (LYM), and B lymphocyte percentage (B%) between the PPDM‐A group and the PPNG‐A group (Table 2). Compared to the PPNG‐A group, the CD64 index, CD10^−^NEUT (%), LYM, and B% levels were significantly higher in the PPDM‐A group, while the CD10^+^NEUT (%) level was significantly lower (all *p* < 0.05). There were no significant differences in other indexes (*p* > 0.05).

**TABLE 2:** Comparison of surface expression of neutrophils and monocyte and lymphocyte subsets between PPDM‐A and PPNG‐A.

Item	PPNG‐A (*N* = 220)	PPDM‐A (*N* = 88)	*Z*	*p*
	(M, IQR)			
Age	52, 27	42, 14	−3.779	< 0.001
MFI of CD10‐NEUT	2 555.2, 2 873.6	2 535.1, 3 445.2	−1.457	0.145
MFI of CD64‐NEUT	10 640.7, 15 377.9	12 045.2, 17 149.3	−1.432	0.152
MFI of CD10‐MONO	536.2, 293.0	500.4, 373.4	−0.928	0.353
MFI of CD64‐MONO	90 222.1, 43 281.4	86 392.5, 39 796.8	−1.365	0.172
MFI of HLA‐DR‐MONO	51 143.1, 49 779.2	44 505.4, 43 092.4	−1.440	0.150
CD64 index	2.0, 4.9	4.1, 13.2	−3.146	0.002
HLA‐DR^−^MONO (%)	0.4, 0.5	0.4, 0.5	−1.408	0.159
CD10^−^NEUT (%)	9.8, 31.7	19.8, 52.5	−2.193	0.028
CD10^+^NEUT (%)	61.1, 24.8	51.4, 47.0	−2.524	0.012
LYM (个/ul)	949.0, 831.5	1 211.0, 925.8	−2.407	0.016
CD3^+^ T (%)	71.9, 19.0	69.1, 13.4	−1.672	0.094
CD3^+^CD4^+^CD8^−^ T (%)	42.4, 15.8	40.0, 14.1	−0.786	0.432
CD3^+^CD4^−^CD8^+^ T (%)	25.9, 12.8	25.6, 10.8	−0.869	0.385
DPT (%)	0.3, 0.4	0.4, 0.5	−1.505	0.132
DNT (%)	0.8, 1.2	0.9, 1.1	−1.107	0.268
B (%)	13.6, 9.8	16.9, 9.5	−3.576	< 0.001
NK (%)	10.9, 11.6	12.1, 7.9	−0.032	0.974
NK/T (%)	3.3, 2.6	2.7, 2.5	−1.308	0.191

Abbreviations: DNT, double negetive T lymphocyte; DPT, double positive T lymphocyte; LYM, lymphocytes; MFI, mean fluorescence intensity; MONO, monocytes; NEUT, neutrophils.

[Correction added on 24 January 2025, after first online publication: the data on 2nd to 5th row on first column of Table 2 have been amended.]

### 3.3 The Correlations Among Surface Expression of Neutrophils/Monocyte Cell and Lymphocyte Subsets

**FIGURE 2:**
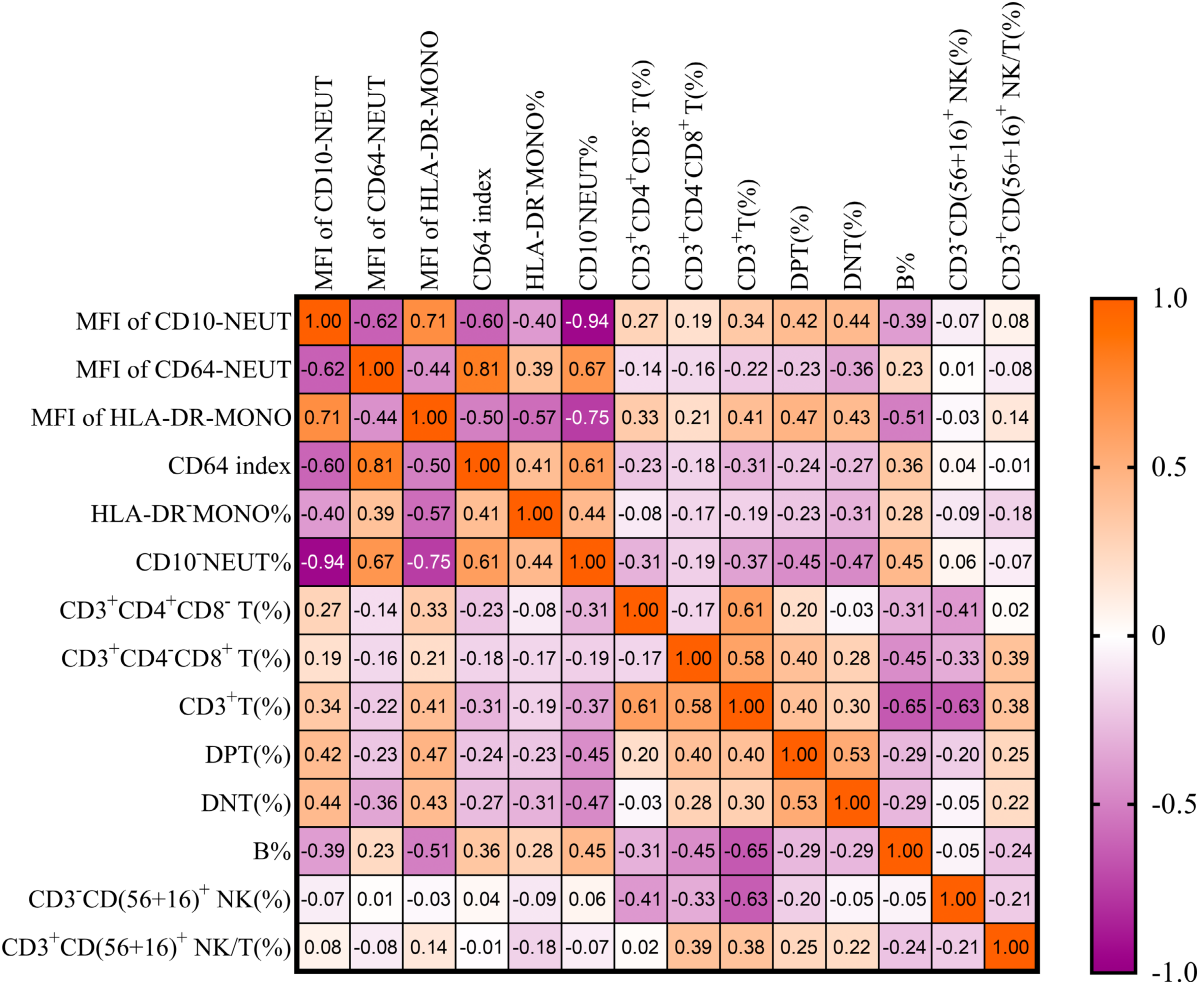
The correlations among surface expression of neutrophils/monocyte cell and lymphocyte subsets.

In the Figure 2, we present the correlation between 14 variables, including granulocyte and monocyte surface markers and lymphocyte subsets. Among the related indexes of neutrophil and monocyte surface expression, only CD10^−^NEUT (%) and the MFI of HLA‐DR‐MONO were moderately correlated with B%. Specifically, there was a moderate positive correlation between CD10^−^NEUT (%) and B% (*r*_s_ = 0.45, *p* < 0.001). Conversely, a moderate negative correlation was observed between the MFI of HLA‐DR‐MONO and B% (*r*_s_ − 0.51, *p* < 0.001). Additionally, there was a strong negative correlation between the MFI of HLA‐DR‐MONO and CD10^−^NEUT (%) (*r*_s_ = −0.75, *p* < 0.001). Both the MFI of CD64‐NEUT and the CD64 index were moderately negatively correlated with the MFI of CD10‐NEUT (*r*_s_ = −0.62 and − 0.60, *p* < 0.001), and moderately positively correlated with CD10^−^NEUT (%) (*r*_s_ = 0.67 and 0.61, *p* < 0.001) (Figure 2).

### 3.4 The Linear Relationship Among CD10 ^−^ NEUT%, MFI of HLA‐DR‐MONO, and B%

**FIGURE 3:**
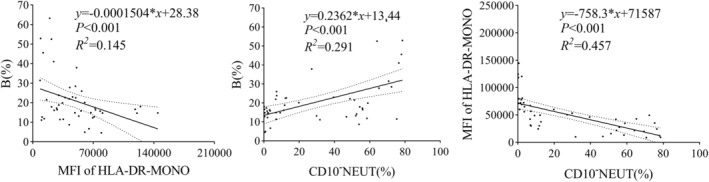
The linear relationship among CD10^−^NEUT%, MFI of HLA‐DR‐MONO and B%.

The MFI of HLA‐DR‐MONO had a negative effect on B% (*R* ^2^ = 0.145, *p* < 0.001), while CD10^−^NEUT (%) had a positive effect on B% (*R* ^2^ = 0.291, *p* < 0.001). Compared to the MFI of HLA‐DR‐MONO, the effect of CD10^−^NEUT (%) on B% was more significant. Additionally, CD10^−^NEUT (%) could explain 45.7% of the variation in the MFI of HLA‐DR‐MONO (*R* ^2^ = 0.457, *p* < 0.001), and the effect was negative (Figure 3).

### 3.5 Influencing Risk Factors of PPDM‐A

**FIGURE 4:**
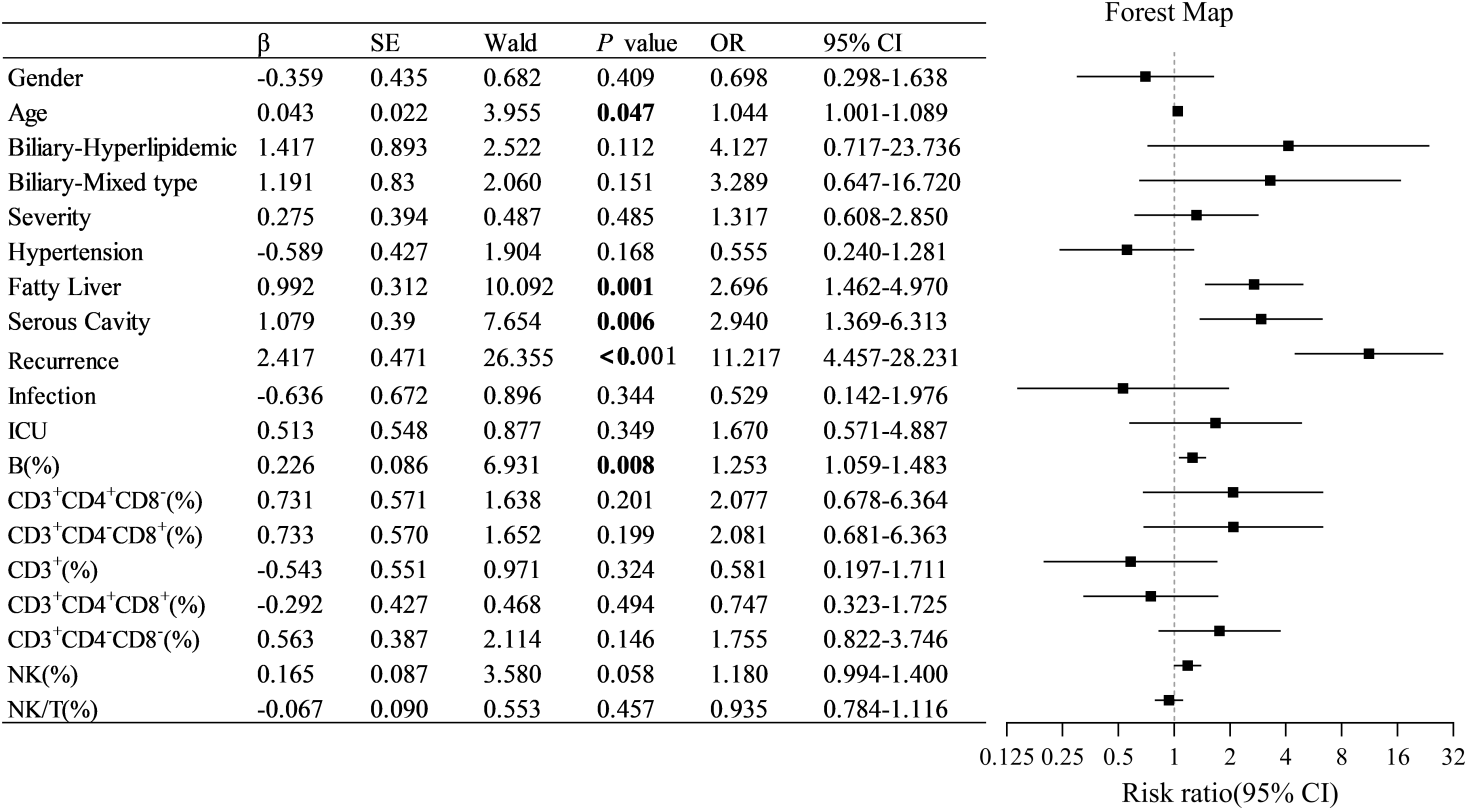
Risk factors of PPDM‐A by multiple logistic regression analysis.

The above clinical features were included in a multiple logistic regression analysis to explore the potential influencing risk factors of PPDM‐A. The results showed that age, fatty liver, serous effusion, recurrent AP, and B% were independent risk factors for the development of PPDM‐A (Figure 4). Among these factors, recurrent AP had the most significant association (*p* < 0.001), followed by fatty liver, serous effusion, and B%. Other lymphocyte subset indicators were not associated with an increased risk of PPDM‐A.

## 4 Discussion

In recent years, an increasing number of studies have focused on the endocrine dysfunction in patients with pancreatic exocrine diseases, particularly the pathogenesis related to PPDM‐A. However, the pathophysiology of PPDM‐A remains complex and not fully understood. Abnormal blood glucose levels are a sign of diabetes in patients with endocrine dysfunction or AP. Once the condition progresses to permanent hyperglycemia, various diabetic complications will gradually emerge, reducing the patient's quality of life. Therefore, identifying the risk factors for glucose metabolism following AP is considered helpful in correcting hyperglycemia and optimizing the treatment strategies for DEP. However, the changes in elevated blood glucose following the onset of AP are subtle and variable, making them difficult to recognize. This may lead to long‐term neglect of blood glucose monitoring and management in AP patients, and even result in misdiagnosis as T2DM.

The reported incidence of PPDM ranges from 5% to 60% in the medical literature. In a meta‐analysis of 15 studies with mixed severity, 23% of patients were newly diagnosed with diabetes after AP [15]. For those with SAP, the prevalence rate can be even higher [16]. A meta‐analysis by Das et al. [15] found that age and obesity were risk factors for PPDM. Our findings also suggest that the incidence of PPDM‐A is 28.6%. Compared with the PPNG‐A group, the PPDM‐A group included younger patients and had a higher proportion of patients with fatty liver, with 72.7% of PPDM‐A patients having hyperlipidemic pancreatitis. Robertson FP et al. [3] analyzed 401 patients in an international registry study and found that the occurrence of PPDM was not an independent predictor of increased length of stay (LOS), ICU admission, or overall mortality. Our data show that, except for the higher incidence of CRRT demand in patients with hyperlipidemic pancreatitis, there was no significant increase in the need for ventilatory support or ICU admission. Yingqi Lv and colleagues demonstrated that the prevalence of newly diagnosed diabetes after the first episode of AP in China is 6.2%. Stress hyperglycemia, hyperlipidemia, non‐alcoholic fatty liver disease (NAFLD) nand recurrent episodes of AP were identified as independent risk factors for PPDM‐A [17]. However, they did not perform further analysis or discussion to investigate changes in relevant indicators.

Diabetes in patients with severe and recurrent AP may result from structural damage to islet β‐cells. As pancreatic necrosis increases, extensive destruction of islet β‐cells and functional failure lead to insufficient insulin secretion [16]. However, even in patients with MAP (without necrosis), the risk of diabetes increases [2]. Furthermore, there is no correlation between the severity of pancreatitis and the risk of diabetes [17]. This suggests that the occurrence of diabetes may be related to other mechanisms, such as local or systemic inflammation, insulin resistance, and others. Therefore, although pancreatic necrosis and recurrent episodes of AP are the most reliable risk factors for diabetes [2], these conditions are not necessary for PPDM‐A [18]. PPDM‐A shows insulin resistance characteristics similar to those of T2DM [4]. Additionally, systemic inflammation associated with pancreatitis may affect blood glucose levels by causing changes in specific immune cell subsets. In our comparison of lymphocyte subsets between the PPDM‐A group and the PPNG‐A group, we found that B% and LYM were significantly higher in the PPDM‐A group than in the PPNG‐A group, with a statistically significant difference (*p* < 0.05). A study by C. Hara Cde et al. [19] also found that lymphocyte levels tend to increase slightly in patients with diabetes, which is consistent with our findings. Increasing evidence suggests that B cells play a significant role in the development of insulin resistance in diabetes. One study reported that B cells could influence Th17 proliferation and the production of pro‐inflammatory cytokines in patients with T2DM [20]. Additionally, B cells have been shown to play a central role in autoimmune diabetes. P. Wu et al. [12] demonstrated that islet cell antibodies (ICA) and IAA are associated with various B cell subsets. This suggests that imbalanced B cell development is involved in the process of islet autoimmunity in diabetes. Moreover, plasma cells (PB) were negativelycorrelated with fasting C‐peptide (FCP), further indicating that B cells might contribute to β‐cell destruction in patients with diabetes. B cell‐targeted therapy had also been successfully used in treating T1DM [21]. However, these studies are based on T1DM, T2DM, and autoimmune diabetes. Currently, no studies have investigated the immune mechanisms of PPDM‐A. Our results suggest that in the context of AP, changes in lymphocyte subsets (B cells (%) and LYM) are associated with the onset of diabetes.

AP is considered a cascade of immune responses triggered by acinar cell necrosis [22]. The AP process is accompanied by the infiltration of innate immune cells, primarily macrophages and neutrophils [23]. The expression of CD10 is closely related to neutrophil maturation and occurs only at the final stage of neutrophil development [24]. CD10^−^NEUT (%) can be used to assess the number of bone marrow neutrophils released into the bloodstream during acute inflammatory stimulation [25]. Studies by K. Venkatesh et al. [26] have shown that lymphopenia in patients with AP is usually associated with an increase in neutrophil count. Interestingly, in our study, the opposite immune response was observed in patients with PPDM‐A. We found that CD10^−^NEUT (%) was positively correlated with B% and LYM in patients with PPDM‐A. Immunophenotypic analysis revealed that the CD64 index, CD10^−^NEUT (%), LYM, and B% in PPDM‐A patients were significantly higher than those in the PPNG‐A group, while CD10^+^NEUT (%) was significantly lower. These differences suggest that abnormal activation or regulatory imbalance of the immune system may be present in patients with PPDM‐A.

The increase in CD10^−^NEUT and immature granulocyte levels may be related to the presence of myeloid‐derived suppressor cells (MDSCs) in peripheral blood, a phenomenon observed in septic patients [27]. The proportion of MDSCs in the peripheral blood of patients with AP increased and was positively correlated with the severity of AP. MDSCs from SAP patients exhibited a significant inhibitory effect on CD3^+^ T cells [28]. In our study, we found that CD3^+^ T (%), CD3^+^CD4^+^CD8^−^ T (%), and CD3^+^CD4^−^CD8^+^ T (%) in the PPDM‐A group were lower than those in the PPNG‐A group, although the differences were not statistically significant (*p* > 0.05), possibly due to the need for a larger sample size. However, it is noteworthy that while CD10^−^NEUT (%) was not directly associated with the development of PPDM‐A, it was positively correlated with B%. B% is an independent risk factor for PPDM‐A. Correlation analysis showed that both CD10^−^NEUT (%) and the MFI of HLA‐DR‐MONO were related to B%. The linear regression coefficient between CD10^−^NEUT (%) and B% was high (*R* ^2^ = 0.291, *p* < 0.001), suggesting that CD10^−^NEUT may play a role in regulating B cell activation. Given the immunosuppressive characteristics of MDSCs under various pathological conditions, the immature state of CD10^−^NEUT may have similar functions. This immunosuppression can be mediated through various mechanisms, including direct cell contact, inhibition of cytokine secretion, and regulation of metabolic pathways. These mechanisms may lead to decreased T cell activity, thereby affecting the immune response. The systemic immune stress response is triggered by the self‐digestion of pancreatic tissue, leading to B cell proliferation and the secretion of inflammatory mediators [29]. These mediators not only promote inflammation but also inhibit T cell function, explaining the reduction in total T lymphocytes and the increase in both B% and total lymphocytes in the PPDM‐A group. This suggests that the relationship between CD10^−^NEUT(%) and B% may play an important role in PPDM‐A.

CD10 and HLA‐DR are two biomarkers that show potential clinical applications in various inflammatory and immune‐mediated diseases [30]. Our correlation analysis revealed a strong negative correlation between CD10^−^NEUT (%) and the MFI of HLA‐DR‐MONO (*r*_s_ = −0.750, *p* < 0.001), and linear regression analysis also suggested a strong linear relationship between these two parameters (*R* ^2^ = 0.457, *p* < 0.001). This suggests that CD10^−^NEUT may play a key role in regulating monocyte activation. In acute inflammation or infection, the release of immature neutrophils is often accompanied by immunomodulatory mechanisms. HLA‐DR is a crucial molecule on the surface of monocytes responsible for antigen presentation. Its downregulation is typically associated with immunosuppression, which may represent the body's attempt to prevent excessive inflammation [30]. Repurposing drugs and giving various vitamins as E and D as prophylactic with modulatory effect with positive impact on inflammation and lymphocytes [9]. The findings of our study are consistent with the pathophysiological mechanisms of AP.

## 5 Conclusion

In summary, Our study has first confirmed the correlation between PPDM‐A and lymphocyte subsets and CD10^−^NEUT%. Furthermore we indicated that age, fatty liver, serous effusion, recurrent AP, and B% were independent risk factors for PPDM‐A. The mechanism of granulocyte and monocyte surface markers and B lymphocytes on PPDM‐A is worthy of study. The mechanism of granulocyte and monocyte surface markers and B lymphocytes on glucose metabolism disorder after AP is worthy of further study. This would help clarify the pathogenesis of PPDM‐A at the cellular level and potentially provide new strategies for immunotherapy and even disease prevention.

### 5.1 Recommendation and Future Perspectives

Of course, there are still some limitations in our research. Although this study reveals important differences between PPDM‐A and PPNG‐A, the small sample size may affect the generalizability of the results. While we suggest that CD10^−^NEUT may play a role in regulating B%, the *R* ^2^ value is not high, indicating that other factors may influence B%. In the future, more potential variables or mechanisms should be considered to fully explain the relationship between these two variables. Additionally, since the fine classification of B lymphocytes was not assessed in this study, the function of B lymphocytes was not evaluated. Future research may focus on analyzing the subsets of B lymphocytes at the cellular level in patients' peripheral blood and explore the regulatory mechanisms behind changes in B cell subsets. This would help clarify the pathogenesis of PPDM‐A at the cellular level and potentially provide new strategies for immunotherapy and even disease prevention.

## Author Contributions

Xiaoyan Lin designed the research and revised the draft and was in charge of specimen testing. Xiaoyan Lin and Xiaoling Li analyzed the data, and wrote. Xiaoling Li was responsible for the chart modification. Huiheng Liu, Junsheng Wang collected and organized the clinical information. Xiaoyan Lin and Huiheng Liu overall grasped the quality of the article. All authors have read and approved the manuscript.

## Ethics Statement

This study was approved by the Institutional Ethics Committee of Zhongshan Hospital, School of Medicine, Xiamen University, and complied with national legislations and the Declaration of Helsinki guidelines (xmzsyyky‐2023‐063).

## Consent

The subjects included in this study were adults, and all of the subjects provided written informed consent in accordance with the institutional guidelines prior to the study.

## Conflicts of Interest

The authors declare no conflicts of interest.

## Data Availability

The data related to this manuscript will be made available upon request.
